# Yangjing Capsule Can Improve the Function of the Testicular Angiogenesis through Activating VEGFA/eNOS Signaling Pathway

**DOI:** 10.1155/2020/1957267

**Published:** 2020-04-25

**Authors:** Baofang Jin, Dalin Sun, Weihang Dong, Bing Chen, Weimin Deng, Bin Cai, Yugui Cui, Yihan Jin, Jianguo Liu, Li Tong, Ping Wu

**Affiliations:** ^1^Andrology Department of Integrative Medicine, Zhongda Hospital, School of Medicine, Southeast University, Nanjing 210009, China; ^2^Medical College, Qinghai University, Xi'ning 810001, China; ^3^Department of Andrology, Yuncheng Hospital of Traditional Chinese Medicine, Yuncheng 044000, China; ^4^State Key Laboratory of Reproductive Medicine, Clinical Center of Reproductive Medicine, First Affiliated Hospital, Nanjing Medical University, Nanjing 210029, China; ^5^Department of Andrology, Shaanxi Provincial Hospital of Traditional Chinese Medicine, Xi'an 710003, China

## Abstract

**Background:**

The testicular microcirculation was an important aspect of testicular physiology and it offered a stable environment for the transport of nutrients and secretary products in the testis. Yangjing capsule (YC), a traditional Chinese compound herbal prescription, has been proved as an effective drug to ameliorate spermatogenesis, promote testosterone synthesis in vivo, and cure spermatogenesis in clinical practice.

**Objective:**

This study was aimed at understanding the potential mechanisms of YC exerting angiogenic effects in the mouse spermatogenesis dysfunction model induced by cyclophosphamide (CP) and MLTC-1 cells.

**Materials and Methods:**

Balb/c mice were randomly divided into five groups: control, CP, CP plus YC (630 mg/kg), CP plus YC (1260 mg/kg), and CP plus YC (2520 mg/kg). After 30 days, mice were sacrificed and the expressions of endothelial marker CD34+, angiogenic marker VEGFA, VEGFR1, VEGFR2, and eNOS in the testes of the mice were examined; moreover, Leydig cell line MLTC-1 cells were cultured and treated with different concentrations of YC extracts (YCE), and the expressions of VEGFA, VEGFR1, VEGFR2, and eNOS, as well as the secretion of NO, were evaluated.

**Results:**

We observed that YC significantly increased the expressions of VEGFA, VEGFR1, VEGFR2, and eNOS in testes of CP-treated mice; moreover, YCE has led to increased expressions of VEGFA, VEGFR1, VEGFR2, and eNOS and secretion of NO in MLTC-1 *in vitro*. These data suggested that the YC might be an alternative treatment for the dysfunction of testicular microcirculation by promoting the angiogenesis in the testis.

## 1. Introduction

The testicular microcirculation was an important aspect of testicular physiology. Functionally, it offered stable environment for transport of nutrients and secretary products in the testis [1, 2]. Angiogenesis represents the formation of new vessels from existing vasculature. It is necessary to have adequate and appropriate amount of angiogenesis in testis. Either impaired or excessive angiogenesis was associated with the abnormal pathophysiologic alterations of the testis which can further lead to abnormalities in microcirculation and impaired the testicular functions such as spermatogenesis and hormonal and paracrine control [3, 4].

The vascular endothelial growth factor (VEGF) is a well-known angiogenic factor [5]. It was regarded as important candidates for the regulation of physiological and pathophysiological angiogenesis [6]. The VEGF was also an endothelium-specific secreted protein that potently stimulated vasodilation and microvascular hyperpermeability [7, 8]. Among the angiogenic factors, VEGF is a multifunctional protein, and it played a key role in new blood vessel formation. Previously, it had been shown that a member of the VEGF family, vascular endothelial growth factor A (VEGFA) was a powerful mitogen for human dermal microvascular endothelial cells that expressed both VEGFR1 and VEGFR2 *in vitro* [9]. In addition, VEGFA could stimulate the endothelial cell of microvessels to proliferate, migrate, and change their gene expression pattern [10]. More and more research studies had proved the important role of VEGFA in microcirculation [11, 12].

The Yangjing capsule (YC) is a traditional Chinese compound herbal preparation. It consisted of *Herba Epimedii Brevicornus*, *Rhizoma Polygonati Sibirici*, *Placenta Hominis*, *Radix Rehmanniae Preparata*, *Angelica Sinensis*, and other components [13]. YC had been used to treat male infertility and sexual dysfunction for many years in China. A previous study suggested that YC could enhance sperm motility and testosterone synthesis for infertile men [14]. Another study reported that YC could ameliorate spermatogenesis in male mice induced by CP; hence, YC may be an efficient drug to improve spermatogenesis in patient [13]. Some components in YC were considered to regulate angiogenesis in vitro and in vivo. For instance, *Angelica Sinensis* exerted antiangiogenesis effects in human umbilical vein endothelial cells [15]. Steroidogenesis acute regulatory protein (StAR), another component in YC, was considered to be involved in Leydig cell angiogenesis [16]. However, the effects of YC on the angiogenesis in testis remain unclear.

In this study, we performed *in vivo* analysis to explore the roles of YC in regulating the angiogenesis in the testis of cyclophosphamide- (CP-) induced mouse spermatogenesis dysfunction model, and we also determined the effect of YC on Leydig cell line MLTC-1 cells *in vitro*. Our data may provide novel evidence for the use of YC as an alternative method to improve the function of the testicular microcirculation by promoting the angiogenesis in the testis.

## 2. Materials and Methods

### 2.1. Chemicals and Drugs

The PrimeScript RT Master Mix and SYBR Green PCR Master Mix reagent kits were purchased from TaKaRa (TaKaRa Biotechnology, Dalian, China). The primers were synthesized by Invitrogen Life Tech (Carlsbad, CA, USA). The protein assay kit was obtained from Beyotime (Beyotime Institute of Biotechnology, Shanghai, China). The cyclophosphamide was purchased from Pude Medicine Co. Ltd. (Nanjing, China). The RPMI 1640 medium and fetal bovine serum (FBS) were purchased from GIBCO (Grand Island, NY, USA).

### 2.2. Preparation of the YC

The YC was obtained from Nanjing General Hospital of Nanjing Military Region (Nanjing, China). It was composed of 11 traditional Chinese drugs: *Radix Rehmanniae Preparata*, *Semen Astragali Complanati*, *Herba Epimedii Brevicornus*, *Mongolici*, *Placenta Hominis, Radix Angelicae Sinensis*, *Hirudo, Rhizoma Polygonati Sibirici, Radix Astragali*, *Semen Litchi, Semen Vaccariae Segetalis*, and *Concha Ostreae* (*calcined*). The YC extract was acquired according to the methods described previously [17].

### 2.3. Animals and Treatments

The mature male (8- to 10-week-old) Balb/c mice (25 ± 2 g) were purchased from Nanjing University (Nanjing, China). Mice were fed with standard food and water ad libitum under standard laboratory conditions. After adapting to the environment for a week, the mice were randomly divided into five groups (10 mice/group): control, CP, CP plus YC (630 mg/kg), CP plus YC (1260 mg/kg), and CP plus YC (2520 mg/kg). For the first 7 days, the mice of CP and YC groups were injected with CP once a day since the first day of the experiment. After 30 days, the mice in the YC treatment groups were administered with YC suspension via gavage once a day. The general health of the animals was assessed daily. Finally, the fresh testis frozen in liquid nitrogen was used for the isolation of RNA and extraction of proteins. This study has been approved by the Animal Ethics Committee of Southeast University.

### 2.4. Immunohistochemistry

On day 30, mice were sacrificed, and the testes were collected, fixed with formalin, embedded with paraffin, and sectioned into 6–8 *μ*m slices. To examine the presence of CD34 in different samples, the sections were treated with the citrate buffer at 100°C for 1 h and then incubated with the anti-CD34 antibodies (ab81289, 1 : 100, Abcam, Cambridge, USA) at 4°C overnight. The following day, the sections were incubated with the secondary antibodies for 1 h and incubated with streptavidin-horseradish peroxidase (SA10001, 1 : 1000, Thermo Fisher Scientific Inc., USA) for 30 min at room temperature. Finally, the sections were stained with diaminobenzidine (DAB), and the images were visualized by microscopy.

### 2.5. Cell Culture

MLTC-1 cells were obtained from the Cell Institute of Shanghai (Shanghai, China). MLTC-1 cells were cultured in RPMI-1640 containing 10% fetal bovine serum in 5% CO_2_ at 37°C. According to a previous study, the cells were treated with 0, 0.01, 0.1, and 1 mg/mL YC extract (YCE) and incubated for up to 24 h in a humidified atmosphere of 5% CO_2_ at 37°C [13].

### 2.6. RNA Extraction and qRT-PCR

Total RNA was extracted with TRIzol reagent (Invitrogen, Carlsbad, CA, USA) according to the manufacturer's protocol. The qRT-PCR assays were performed to detect the expressions of VEGFA, VEGFR1, and VEGFR2 using the PrimeScript RT reagent Kit and SYBR Premix Ex Taq (TaKaRa, Dalian, China) according to the manufacturer's instruction. The PCR primers were designed as follows: VEGFA sense 5′-GCAGGCCGTGGAGTGTGA-3′ and VEGFA reverse 5′-CTACCGCATTTTCTGCATCCT-3′, VEGFR1 primer sense 5′- GGTATCCCTCAACCTACA-3′ and VEGFR1 primer reverse 5′-CCACAGTCCCAACTTTATT-3′, VEGFR2 primer sense 5′-ACTGTCATCCTTACCAATCCCA-3′ and VEGFR2 primer reverse 5′-ATCTGGGGTGGGACATACAC-3′, and GAPDH primer sense 5′-ACGGCTACCGTGATCGAAG-3′ and GAPDH primer reverse 5′- ATCTGGGGTGGGACATACAC-3′. The PCRs were conducted at 95°C for 30 s and followed by 40 cycles of 95°C for 30 s and 59°C for 35 s and 60°C for 30 s in the ABI 7300 Real-Time PCR system (Applied Biosystems, Foster City, CA, USA). We used the 2^−ΔΔ*Ct*^ method to calculate the relative abundance of the target mRNAs. GAPDH has been applied as the internal control.

### 2.7. Western Blot Analysis

The total proteins isolated from testes of the mice and the MLTC-1 cells were obtained following the standard procedures and quantified by using the bicinchoninic acid protein assay (Beyotime, Shanghai, China). The proteins were separated by 12% SDS-polyacrylamide gel electrophoresis and then transferred to polyvinylidene fluoride (PVDF) membranes (Millipore) and incubated with the rabbit anti-VEGFA (ab46154, 1 : 1000, Abcam, Cambridge, USA), rabbit anti-VEGFR1 (ab32152, 1 : 100, Abcam, Cambridge, USA), rabbit anti-VEGFR2 (ab5473, 1 : 100, Abcam, Cambridge, USA), rabbit anti-eNOS (ab5589, 1 : 100, Abcam, Cambridge, USA), and rabbit anti-GAPDH (ab9485, 1 : 2500, Abcam, Cambridge, USA) overnight at 4°C. On day 2, after washing with TBS three times, the membranes were incubated with goat anti-rabbit HRP-conjugated secondary antibodies (ab6721, 1 : 5000, Abcam, Cambridge, USA) at 37°C for 1 h. The relative protein levels in each sample were normalized to the levels of GAPDH to standardize the variations.

### 2.8. Determination of the Content of NO

The content of NO in the cell culture supernatant was determined by a commercially available kit (purchased from Beyotime, Shanghai, China) according to the manufacturer's instructions.

### 2.9. Statistical Analysis

Each cell experiment was repeated three times, and all data are represented as mean ± standard deviation (SD). Analysis of variance (ANOVA) has been applied for the comparisons among multiple groups. *P* values less than 0.05 were considered statistically significant.

## 3. Results

### 3.1. The Effect of YC on Expression of CD34 in Mice Testes

**Figure 1:**
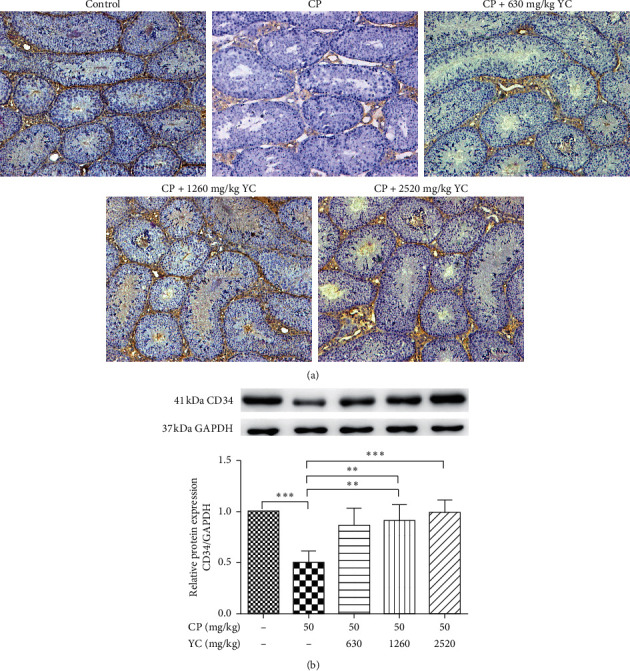
The effect of YC on the expression of CD34+ in testes of mice with different treatments. (a) Images of the IHC staining results (×100) and (b) WB results. ^*∗*^*P* < 0.05, ^*∗∗*^*P* < 0.01, ^*∗∗∗*^*P* < 0.001. CP: cyclophosphamide; SD: standard deviation; YC: Yangjing capsule.

First, we examined the effect of the YC on the angiogenesis in mice testis by comparing the expression of CD34 in different groups using IHC and WB methods. As shown in Figure 1(a), the density of blood vessels was significantly decreased in the CP group and the structure of testicular vasculature was severely damaged, compared with the control group; after YC treatment, the density of blood vessels was increased and the structure damage was partially reversed in a concentration-dependent manner (*P* < 0.01); on the other hand, YC treatment induced presence in the expression of CD34 in mice testes (Figure 1(b), *P* < 0.05) compared with the CP group, and the angiogenic effect of 2520 mg/kg YC was more significant (*P* < 0.01).

### 3.2. The Effect of YC on the Expressions of VEGFA, VEGFR1, and VEGFR2 in Mice Testes

**Figure 2:**
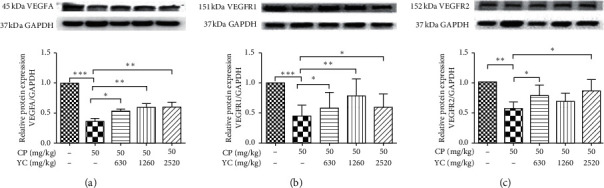
The effects of YC on the expression of VEGFA, VEGFR1, and VEGFR2 in the testes of mice with different treatments. Data given are the mean ± SD. ^*∗*^*P* < 0.05, ^*∗∗*^*P* < 0.01, ^*∗∗∗*^*P* < 0.001. CP: cyclophosphamide; SD: standard deviation; YC: Yangjing capsule.

Moreover, the levels of angiogenic factors VEGFA, VEGFR1, and VEGFR2 in the testes of mice with different treatments were also evaluated. It was observed that CP induced marked decrease in the expressions of VEGFA, VEGFR1, and VEGFR2 compared with the control group, and different concentrations of YC treatment increased the expressions of VEGFA, VEGFR1, and VEGFR2 compared with the model group (Figure 2).

### 3.3. The Effect of YC on the Expression Level of eNOS in Mice Testes

**Figure 3:**
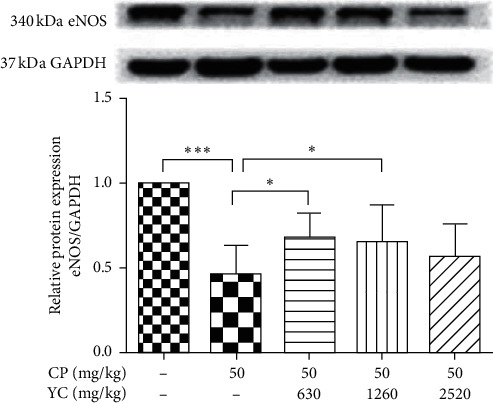
The effects of YC on the expression of eNOS in the testes of spermatogenesis dysfunction mice induced by CP. Data given are the mean ± SD. ^*∗*^*P* < 0.05, ^*∗∗*^*P* < 0.01, ^*∗∗∗*^*P* < 0.001. CP: cyclophosphamide; SD: standard deviation; YC: Yangjing capsule.

eNOS has being known as a downstream enzyme of VEGF. Next, to further examine the angiogenic effects of YC in mice testes, we analyzed the expression profiles of eNOS in the testis of mice with different treatments. As shown in Figure 3, CP has led to decreased expression of eNOS in testes of mice, while on the other hand, 630 mg/kg and 1260 mg/kg YC induced a significant increase in the expression of eNOS in testes of the CP-treated mice (*P* < 0.05); moreover, 2520 mg/kg YC had no significant effect on the expression of eNOS in testes of CP-treated rats.

### 3.4. The Effect of YCE on the Expressions of VEGFA, VEGFR1, and VEGFR2 in MLTC-1 Cells

**Figure 4:**
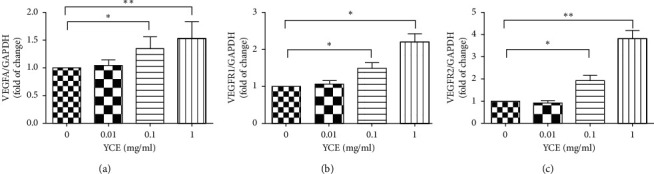
The effects of YCE on the mRNA expression of VEGFA, VEGFR1, and VEGFR2 in MLTC-1 cells. Data given are the mean ± SD. ^*∗*^*P* < 0.05, ^*∗∗*^*P* < 0.01, ^*∗∗∗*^*P* < 0.001. CP: cyclophosphamide; SD: standard deviation; YCE: Yangjing capsule extract.

**Figure 5:**
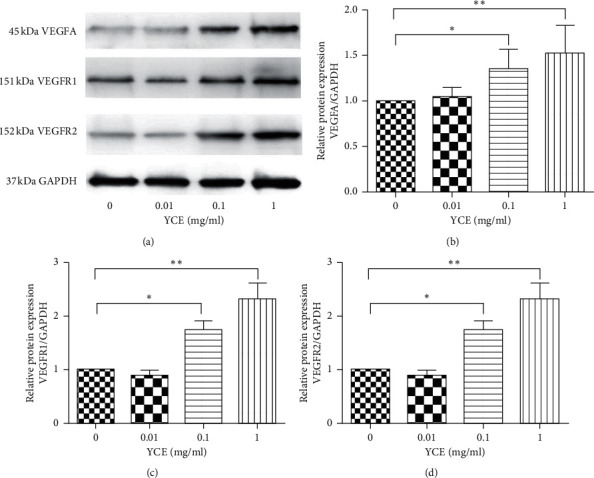
The effects of YCE on the protein expressions of VEGFA, VEGFR1, and VEGFR2 in MLTC-1 cells. Data given are the mean ± SD. ^*∗*^*P* < 0.05, ^*∗∗*^*P* < 0.01, ^*∗∗∗*^*P* < 0.001. CP: cyclophosphamide; SD: standard deviation; YCE: Yangjing capsule extract.

To further explore the underlying mechanism of YC-induced angiogenic effects, the MLTC-1 cells were cultured and treated with different concentrations (0.01, 0.1, and 1 mg/mL) of YC extracts (YCE), and the expressions of VEGFA, VEGFR1, and VEGFR2 were examined by quantitative real-time PCR and WB methods. We observed that 0.1 and 1 mg/mL YCE significantly increased the mRNA expression of VEGFA, VEGFR1, and VEGFR2 in a dose-dependent manner (Figure 4); moreover, results of WB analysis revealed that 0.1 and 1 mg/mL YCE markedly increased the protein expressions of VEGFA, VEGFR1, and VEGFR2 in a dose-dependent manner (Figure 5). On the other hand, 0.01 mg/mL YCE had no significant effects on the expressions of VEGFA, VEGFR1, and VEGFR2 in MLTC-1 cells.

### 3.5. The Effect of YCE on the Secretion of NO and Expression of eNOS in MLTC-1 Cells

**Figure 6:**
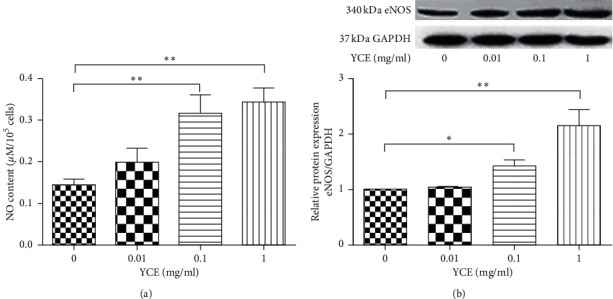
The effects of YCE on the secretion of NO and expression of eNOS in MLTC-1 cells. Data given are the mean ± SD. (a) Content of NO in the cell culture supernatant of different treatments; (b) expression of eNOS in different groups. ^*∗*^*P* < 0.05, ^*∗∗*^*P* < 0.01, ^*∗∗∗*^*P* < 0.001. CP: cyclophosphamide; SD: standard deviation; YCE: Yangjing capsule extract.

Finally, the secretion of NO and expression of eNOS in MLTC-1 cells of different treatments were also evaluated. As shown in Figure 6, 0.1 and 1 mg/mL YCE significantly increased the secretion of NO in the cell culture supernatant of MLTC-1 cells in a dose-dependent manner (Figure 6(a)); furthermore, 0.1 and 1 mg/mL YCE also lead to increased expressions of eNOS in a dose-dependent manner (*P* < 0.05). On the other hand, 0.01 mg/mL YCE had no significant effects on the secretion of NO and expression of eNOS in MLTC-1 cells.

## 4. Discussion

In the present study, we found the density of blood vessels was significantly decreased and the structure of testicular vasculature was severely damaged when exposed to CP; meanwhile, the decrease and damage could be partially reversed by adding YC in a concentration-dependent manner. Moreover, the presence of CD34 (an endothelial marker) was observed after YC treatment as well; moreover, we also observed that the extract of YC can increase the expression of VEGFA, VEGF receptors, and eNOS and increase the production of NO in Leydig cell line MLTC-1 cells. Our results suggested that YC may improve the angiogenesis in testis.

The protective roles of YC in male reproductive diseases have been discussed in several studies conducted by our research group. We previously reported that YC can increase the secretion of testosterone in MLTC-1 cells and may be a potential an alternative method for the treatment of insufficient testosterone relate diseases [13, 14]; moreover, YCE has been shown to activate the PI3K pathway and induced the self-renewal of the GC-1 spg cells [17, 18]; furthermore, in a very recent study, we observed that YC can inhibit the apoptosis of the MLTC-1 cells and via promoting the expression of StAR [16]. Meanwhile, in testis, the vasculature is of great importance because pituitary gonadotropins are delivered by testicular vasculature to support the process of spermatogenesis [19]; however, whether YC can promote testicular microcirculation or the angiogenesis in testis is still unclear. In the present study, we first observed that CP treatment caused damage to the testicular vasculature, and on the other hand, YC protected the testicular vasculature in the testis of CP-treated spermatogenesis mice. Taken together, we proposed that YC may exert angiogenic effects in the testis of spermatogenesis dysfunction mouse, which may further lead to the regeneration of the testicular vasculature and alleviate the condition of spermatogenesis dysfunction.

VEGFA is a specific endothelium protein that potently stimulated angiogenesis, vasodilation, and microvascular hyperpermeability [11, 12, 20]. Functionally, it could alter its gene expression level to promote proliferation and migration for the endothelial cells. In testis, besides the angiogenic function, VEGFA can also stimulate the proliferation and testosterone secretion of Leydig cells [21]; moreover, VEGFA has been observed to regulate spermatogenesis via promoting the self-renewal, differentiation, and proliferation of the spermatogonial cells [22, 23]. In the current study, we observed that YC increased the expression of VEGFA and VEGF receptors in testes of CP-treated mice, suggesting that YC may promote angiogenesis in testis via regulating the expression of VEGFA; furthermore, Leydig cells secrete a number of growth factors, including VEGFA [24, 25]. To further explore the angiogenic effect of YC in testes, the *in vitro* effect of YC extract on the expression of VEGFA and VEGF receptors was also evaluated. Consistent with the *in vivo* observation, 0.1 and 1 mg/mL YCE induced increased expression of VEGFA and VEGFR on both mRNA and protein levels in Leydig cell line MLTC-1 cells *in vitro*. Taken together, these results suggested that YC can increase the expression of VEGF both in vitro and in vivo, which may induce its angiogenic function in testis.

Nitric oxide (NO) was a pleiotropic molecule critical to several physiological and pathological processes (reference). NO was synthesized from the amino acid L-arginine by either the constitutive endothelial NOS (eNOS) or the inducible NOS (iNOS) [26]. It has been reported in previous studies that VEGF can increase the expression of eNOS and secretion of NO [7, 8, 27, 28]. In the current study, we observed that 630 mg/kg and 1260 mg/kg YC significantly increased in the expression of eNOS in testes of the CP-treated mice *in vivo*, and 0.1 and 1 mg/mL YCE also significantly increased the secretion of NO and the expressions of eNOS in MLTC-1 cells *in vitro*. Taken together, these results indicated that YC may activate the VEGFA/eNOS signaling pathway in testes of mice and MLTC-1 cells to exert its therapeutic effects.

## 5. Conclusion

In conclusion, we found that the Yangjing capsule may exert angiogenic effects in testes of mice spermatogenesis dysfunction models and MLTC-1 cells via activating VEGFA/eNOS signaling pathway. Our data suggested that Yangjing capsule may be an alternative medicine for the treatment of male infertility or hypogonadism diseases.

## 6. Additional Points

YC significantly increased the expressions of VEGFA, VEGFR1, VEGFR2, and eNOS in testes of CP-treated mice. YCE has led to increased expressions of VEGFA, VEGFR1, VEGFR2, and eNOS and secretion of NO in MLTC-1 *in vitro*. YC might be an alternative treatment for the dysfunction of testicular microcirculation by promoting the angiogenesis in the testis.

## Data Availability

All relevant data are within the paper and its Supporting Information files.
